# Latent Salinity Stress Detection in *Opuntia ficus-indica* Using Hyperspectral Imaging and a 3D-CNN Framework

**DOI:** 10.3390/s26123641

**Published:** 2026-06-07

**Authors:** Juan Arredondo-Valdez, Horacio Abdiel Rodríguez-Garza, Héctor Flores-Breceda, Zayd Eliud Rangel-Nava, Néstor Everardo Aranda-Ledesma, Jesús Rodolfo Valenzuela-García, Moisés Hinojosa-Rivera, Ajay Kumar, Urbano Luna-Maldonado, Alejandro Isabel Luna-Maldonado

**Affiliations:** 1 Department of Agricultural and Food Engineering, Faculty of Agronomy, Autonomous University of Nuevo Leon, Francisco Villa S/N, Ex-Hacienda El Canadá, General Escobedo 66050, Nuevo Leon, Mexico; juan.arredondovld@uanl.edu.mx (J.A.-V.); abdiel.rodriguezgrz@uanl.edu.mx (H.A.R.-G.); hector.floresbrc@uanl.edu.mx (H.F.-B.); zayd.rangelnv@uanl.edu.mx (Z.E.R.-N.); nestor.arandald@uanl.edu.mx (N.E.A.-L.); 2 Department of Agricultural Machinery, Antonio Narro Autonomous Agricultural University, Calzada Antonio Narro 1923, Buenavista, Saltillo 25280, Coahuila, Mexico; jesus.valenzuela@uaaan.edu.mx; 3 Faculty of Mechanical and Electrical Engineering, Autonomous University of Nuevo Leon, Pedro de Alba S/N, Ciudad Universitaria, San Nicolás de los Garza 66455, Nuevo Leon, Mexico; moises.hinojosar@uanl.mx; 4 Department of Biosystems and Agricultural Engineering, Oklahoma State University, Stillwater, OK 74078, USA; ajay.kumar@okstate.edu

**Keywords:** *Opuntia ficus-indica*, 3D-CNN, hyperspectral imaging, salinity stress, precision agriculture, non-destructive phenotyping

## Abstract

Salinity stress remains a major bottleneck for agriculture in arid regions. While *Opuntia ficus-indica* is known for its resilience, its young cladodes maintain a misleadingly healthy visual appearance and stable biomass even under heavy saline pressure, making traditional vegetation indices and standard statistics unreliable for early diagnosis. The objective of this study was to develop a non-destructive phenotyping framework for the early detection of latent salinity stress in young *Opuntia* cladodes. Controlled experiments were conducted using hyperspectral data cubes (400–1000 nm) acquired from plants exposed to six distinct salinity levels ranging from 2 to 21 dS m^−1^. Our methodology integrates these high-dimensional spatial–spectral data with a tailor-made 3D Convolutional Neural Network (3D-CNN). Seven physiological vegetation indices—NDVI, PRI, WI, PSRI, MCARI, SIPI, and NDRE were extracted to track sub-clinical shifts and processed as a volumetric depth dimension within the network to preserve spatial–spectral integrity. The optimized 3D-CNN framework achieved a validation accuracy of 99.7% and a weighted F1-score of 99.1%, delivering 100% precision at critical stress thresholds (13 and 21 dS m^−1^). Spatial confidence maps (Softmax > 0.95) further confirmed the high reliability of the diagnostic output. Requiring a training duration of approximately 8 s, this framework provides a robust basis for precision early-warning irrigation systems to sustain *Opuntia* cultivation in challenging environments.

## 1. Introduction

Mexico serves as the primary center of biodiversity for the genus *Opuntia*, harboring over 100 species predominantly in arid and semi-arid regions. The specialized physiological and morphological adaptations of the prickly pear allow it to thrive under severe water scarcity and extreme temperature fluctuations [1]. The main photosynthetic unit is the cladode, a modified succulent stem that functionally replaces leaves, which have evolved into spines, glochids, and larger structural spines to minimize water loss through Crassulacean Acid Metabolism (CAM) [2,3]. Mexico’s national production of nopal pads reached 868,956 tons in 2021, with Morelos contributing approximately 46.6% of the total, highlighting its economic, nutritional, and industrial significance [4,5]. Beyond food security, *Opuntia’s* mucilage and structural carbohydrates offer promising pathways for developing biodegradable films and bioenergy alternatives, pending industrial optimization [6,7].

Despite its renowned resilience to extreme drought, *Opuntia ficus-indica* remains highly sensitive to soil salinity, an escalating global threat driven by climate change and the over-extraction of groundwater in semi-arid environments [8,9]. In arid regions, brackish water is frequently used for irrigation. However, chronic exposure to sodium chloride (NaCl) triggers osmotic stress and ion toxicity, which severely disrupt cellular homeostasis, reduce photosynthetic efficiency, and impair long-term cladode development [10,11]. Previous studies indicate that soil or irrigation water is considered saline at an electrical conductivity (ECe) as low as 2 dS m^−1^ [12,13]; yet, because *Opuntia* features significant water-storage parenchyma, young cladodes often maintain a misleadingly healthy visual appearance and stable biomass during initial stress phases. This latent buffering effect hinders early visual diagnosis, underscoring the urgent need for non-destructive, sub-clinical monitoring frameworks to sustain crop yields and prevent irreversible tissue damage [13,14].

To address this diagnostic latency, optical remote sensing and proximal phenotyping have emerged as pivotal non-destructive tools in precision agriculture [15]. Vegetation indices (VIs) provide rapid, non-destructive measures of structural and physiological plant status. Traditional indices such as the Normalized Difference Vegetation Index (NDVI) and Water Index (WI) effectively capture macro-level shifts in canopy greenness and tissue hydration, whereas physiological indicators like the Photochemical Reflectance Index (PRI) track subtle, early-stage xanthophyll cycle dynamics and photosynthetic efficiency shifts before chlorophyll degradation manifests visibly [16,17]. While integrating these targeted VIs allows researchers to map invisible physiological responses [18,19], traditional optical sensors often fail to resolve the complex, high-dimensional interactions under multi-level saline stress [20]. To overcome these limitations, high-resolution proximal sensors have gained traction; for instance, recent studies have successfully employed advanced hyperspectral technology to map early ionic and osmotic fluctuations in crops well before external physiological damage occurs [21,22].

Recent advances in Artificial Intelligence (AI) and Machine Learning (ML) have transformed automated crop phenotyping and stress characterization [23,24]. Hyperspectral Imaging (HSI) combined with Deep Learning (DL) enables highly precise stress detection by capturing hundreds of contiguous narrow spectral bands alongside spatial patterns beyond human observation [25]. Nevertheless, extracting meaningful features from high-dimensional hyperspectral data cubes remains a significant computational challenge. Conventional machine learning methods and standard 1D or 2D Convolutional Neural Networks (CNNs) often discard spatial information or compress the spectral dimension, resulting in data redundancy, the “curse of dimensionality,” and reduced classification performance [26]. To address this, multidimensional 3D-CNN architectures have recently revolutionized agricultural computer vision [27]. By applying volumetric convolutions, 3D-CNNs simultaneously preserve and extract concurrent spatial–spectral relationships from raw hyperspectral cubes without requiring aggressive or destructive data compression [28,29].

While multidimensional 3D-CNN architectures have shown success in grain and row-crop classification, their application remains virtually unexplored for evaluating the complex, high-mucilage succulent tissues of CAM species under progressive salinity stress, where traditional 2D optical assessments fail due to latent physiological buffering. To address this critical gap, the aim of this study was to decode NaCl-induced physiological changes in young *Opuntia ficus-indica* cladodes across six salinity levels (2 to 21 dS m^−1^). The primary novelty of this work lies in the development of a unique, non-destructive phenotyping framework that couples a custom-optimized 3D-CNN architecture with a strategic pipeline of seven multi-physiological vegetation indices (NDVI, PRI, WI, PSRI, MCARI, SIPI, and NDRE). The main scientific contribution of this study is two-fold: First, it provides a precise mathematical proof that volumetric spatial–spectral convolutions can effectively capture sub-clinical photosynthetic and water-status degradation in succulent tissues before any visual symptoms manifest. Second, it delivers an ultra-rapid (8-s training), high-precision diagnostic tool (99.7% accuracy) along with spatial confidence mapping. Ultimately, this research bridges the gap between deep learning computer vision and CAM crop physiology, offering a robust, high-throughput benchmark to support precision irrigation and sustainable agriculture in climate-vulnerable arid ecosystems.

## 2. Materials and Methods

### 2.1. Opuntia Cladodes

**Figure 1:**
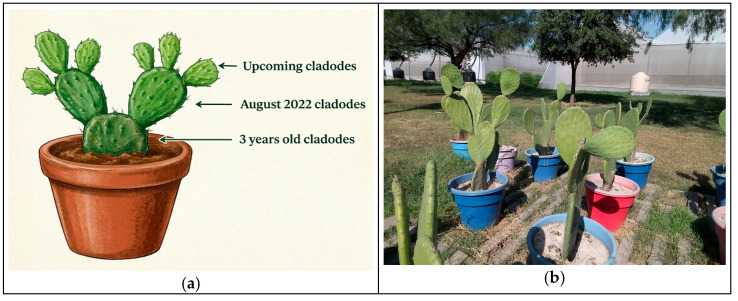
Schematic representation of *Opuntia ficus-indica* cladodes of different ages (**a**) and experimental site (**b**).

Rooted cladodes from three-year-old Villanueva cultivar were planted in 30 individual pots (No. 12 pot, Almoplastic, Celaya, Guanajuato, Mexico), each with a capacity of 22 L (Figure 1). The substrate consisted of clayey loam soil, taken from the top 30 cm of soil at the Alejandra’s Farm in Zuazua, Nuevo León. The soil was sieved and mixed beforehand to ensure uniformity. The pots were placed in a controlled lighting area and watered daily with 200 mL of the corresponding saline solutions, allowing proper drainage of excess water.

A commercial nutrient solution, Grofol 30-20-10 (Agrofarma Mexicana S.A. de C.V., Saltillo, Coahuila, Mexico), was applied once a week. This solution contained nitrogen (20%), phosphorus (30%), potassium (10%), and micronutrients, including sulfur (480 ppm), iron (250 ppm), zinc (250 ppm), magnesium (65 ppm), calcium (65 ppm), copper (65 ppm), boron (65 ppm), cobalt (12 ppm), molybdenum (6 ppm), and phytohormones (12 ppm). The same amount of nutrient solution was applied to all pots, regardless of the salinity treatment.

The experiment was carried out at the Faculty of Agronomy of the Autonomous University of Nuevo León, on the Agricultural Sciences Campus, in General Escobedo, Nuevo León. The geographical location was 25°47′04″ North latitude and 100°17′05″ West longitude, with an altitude of 478 m above sea level. The region has a dry climate (BS0), with annual precipitation ranging from 300 to 600 mm and an average annual temperature between 18 and 24 °C [30].

### 2.2. Experimental Design and Treatments

A completely randomized block design (CRBD) was used, with six saline water treatments, applied to the pots with 5 replicates per treatment. The treatments consisted of NaCl (sodium chloride) solutions, with salinity levels of 2, 5, 10, 13, 18, and 21 dS m^−1^. These NaCl concentrations were representative of the conditions in underground water in the agricultural valleys of northern and western Mexico, where saline concentrations are often high [31].

The NaCl solutions were prepared by dissolving NaCl in distilled water to obtain the desired concentrations, which were verified using a conductivity meter (EC) before application. The solutions were applied every two days, ensuring a constant and controlled supply of saline water. The irrigation frequency was consistent across all treatments.

### 2.3. Monitoring of Environmental Parameters

Environmental parameters were monitored using an automated meteorological station (AcuRite Iris, Lake Geneva, WI, USA) and tensiometers (Irrometer Company Inc., Riverside, CA, USA). Air temperature and relative humidity were recorded with the station sensors, while soil water status was measured using tensiometers installed in the soil. Measurements were taken daily to maintain optimal growing conditions and adjust treatments as needed.

### 2.4. Data Processing and Vegetation Indices

The calculation of vegetation indices (VIs) served as the primary feature extraction layer, allowing for a detailed characterization of the physiological state of *Opuntia ficus-indica* under the varying salinity treatments (2 to 21 dS m^−1^). To mitigate noise and extract physiological signals, seven vegetation indices were calculated using MATLAB 2024b as follows [32]:

### 2.5. Normalized Difference Vegetation Index (NDVI)

NDVI was used to estimate the green biomass and structural vigor of the vegetation, following Peñuelas et al. (1997) [33]:

(1) $$\mathrm{NDVI}=\frac{R_{800}-R_{680}}{R_{800}+R_{680}}$$

### 2.6. Photochemical Reflectance Index (PRI)

The PRI was used to assess photosynthetic stress related to salinity, following the formulation of Peñuelas, Filella, & Gamon (1995) [34]:

(2) $$\mathrm{PRI}=\frac{R_{531}-R_{570}}{R_{531}+R_{570}}$$

where $R_{531}$ and $R_{570}$ are the reflectance values at 531 nm and 570 nm, respectively. Reflectance at these wavelengths was extracted from the hyperspectral data, and the index was calculated using this formula.

### 2.7. Water Index (WI)

This index was used to assess the plants’ water absorption capacity, calculated according to Peñuelas et al. (1997) [33]:

(3) $$\mathrm{WI}=R_{900}-R_{970}$$

where $R_{900}$ and $R_{970}$ are the reflectance values at 900 nm and 970 nm, respectively. Reflectance values were extracted from hyperspectral data, and WI was calculated as the difference between $R_{900}$ and $R_{970}$.

### 2.8. Plant Senescence Reflectance Index (PSRI)

The PSRI was used to indicate the onset of plant senescence, following Merzlyak et al. (1999) [35]:

(4) $$\mathrm{PSRI}=\frac{R_{678}-R_{500}}{R_{750}}$$

where $R_{500}$, $R_{678}$, and $R_{750}$ are the reflectance values at 500 nm, 678 nm, and 750 nm, respectively. Reflectance values were substituted into the formula to calculate PSRI.

Modified Chlorophyll Absorption Reflectance Index (MCARI)

MCARI was used to assess chlorophyll absorption, following Daughtry et al. (2000) [32]:

(5) $$\mathrm{MCARI}=((R_{700}-R_{670})-0.2\cdot (R_{700}-R_{550}))\cdot \frac{R_{700}}{R_{670}}$$

where $R_{550}$, $R_{670}$, and $R_{700}$ are the reflectance values at 550 nm, 670 nm, and 700 nm, respectively. Reflectance values were extracted from the hyperspectral data, and the index was calculated by applying this formula.

### 2.9. SIPI (Structure Insensitive Pigment Index)

SIPI is a spectral vegetation index designed to estimate the ratio between carotenoids and chlorophyll while being relatively insensitive to variations in leaf internal structure. It is widely used in remote sensing applications because it helps emphasize pigment composition rather than structural effects of the leaf. SIPI is primarily used to assess plant stress, detect changes in pigment content (especially the balance between chlorophyll and carotenoids), and evaluate leaf senescence or aging. In practice, it is used for early stress detection, such as drought or heat stress, as well as in plant physiological studies and vegetation pigment assessment, making it a useful indicator of plant health and functioning [36]. The formula is as follows:

(6) $$SIPI=\frac{\left(NIR-Blue\right)}{\left(NIR-Red\right)}=\frac{\left(R_{800}-R_{445}\right)}{{(R}_{800}-R_{680})}$$

### 2.10. NDRE (Normalized Difference Red Edge Index)

NDRE is a vegetation index that uses the red-edge spectral band, which is more sensitive than the traditional red band for detecting variations in chlorophyll, especially in dense vegetation canopies where chlorophyll signals often become saturated. Due to this sensitivity, NDRE is widely used to assess key aspects of plant health, primarily chlorophyll content and plant nitrogen status. This makes it particularly useful for monitoring crop vigor and detecting nutritional deficiencies during mid- to late-growth stages, when other vegetation indices may lose accuracy [33].

(7) $$NDRE=\frac{\left(NIR-RedEdge\right)}{\left(NIR+RedEdge\right)}=\frac{\left(R_{790}-R_{720}\right)}{{(R}_{790}-R_{720})}$$

Chlorophylls were obtained using method described by Delgado-Vargas et al. [37]. First, 25 mL of 80% methanol was added to 0.5 g of pulp sample and homogenized and then centrifuged at 5000 *g* for 5 min at 4 °C, and the absorbance values at 663, 646 and 470 nm were obtained.

Chloropyll *a*: = 12.25A_663_ − 2.29A_646_ (8)

Chloropyll *b* = 21.5A_645_ − 5.21A_663_ (9)

Chloropyll total = 7.15A_663_ + 18.71A_470_ (10)

### 2.11. Acquisition of Hyperspectral Images

Hyperspectral images were acquired using a Pike F-210B camera (Allied Vision Technologies, Stadtroda, Germany) coupled with a V10E spectrograph (Middleton Spectral Vision, Middleton, WI, USA). Data cubes (x, y, λ) were captured over a spectral range of 400–1000 nm at 2.8 nm intervals. 

### 2.12. Image Analysis and Convolutional Neural Network

#### 2.12.1. Data Pre-Processing and Feature Extraction

The acquired hyperspectral cubes were processed and analyzed using MATLAB R2024b [28]. A custom algorithm was developed to extract seven vegetation indices (NDVI, PRI, WI, PSRI and MCARI to characterize the spatial and spectral response of *Opuntia* under salinity stress. These indices were integrated with biochemical and morphological variables, including chlorophyll *a* and *b*, total content, and cladode thickness, to form the input feature space for the deep learning model.

#### 2.12.2. 3D-CNN Architecture

**Figure 2:**
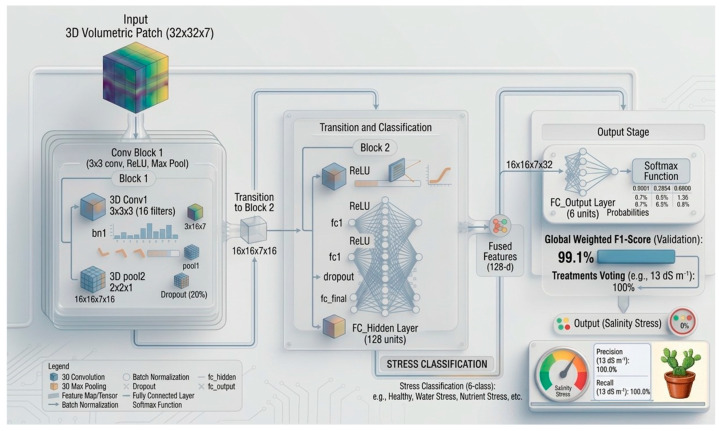
Architectural framework of the optimized 3D-CNN for salinity stress phenotyping. The system processes volumetric input patches (32 x32x7) through dual feature extraction blocks. Block 1 utilizes 3D convolutions (3Dconv1) and spatial–spectral pooling (3Dpool1) with Batch Normalization (bn1) to stabilize the extraction of latent spectral–spatial signatures. Transitioning to Block 2, deeper features are resolved before collapsing the tensor into a 128-dimensional fully connected layer (FC_Hidden Layer), utilizing a high-rate dense dropout (60%) to mitigate spatial overfitting and ensure internal model robustness across independent biological replicates. The Output Stage maps these fused features through the final classification layer (FC_Output Layer) to compute class probabilities via a Softmax activation function, achieving a global weighted F1-score of 99.1% at the patch level and 100% at the plant level via a majority voting consensus. The integrated diagnostic dashboard (bottom right) illustrates the classification precision and recall achieved by the framework within the experimental cohort.

The 3D-CNN architecture was specifically engineered to process the spectral index dimension as a depth component rather than independent channels, thereby preserving the volumetric spatial–spectral structural relationships (Figure 2). The detailed hyperparameter design is itemized in Table 1.

The network consists of a global Z-score standardization layer followed by two hierarchical feature extraction blocks. Block 1 utilizes a 3D convolutional layer with 16 filters and a 3 × 3 × 3 kernel, followed by Batch Normalization (BN) and ReLU activation to stabilize the internal covariate shift. A 3D max-pooling layer (2 × 2 × 1) reduces spatial dimensions while preserving depth representations. Block 2 scales up to 32 filters to resolve highly integrated latent spectral features. To mitigate spatial overfitting, progressive dropout rates of 20%, 30%, and 60% were deployed across the pooling and fully connected layers, forcing the model to learn robust, non-redundant feature representations. The output stage maps the 128-dimensional dense fusion layer onto a 6-unit Softmax activation layer to output discrete class probabilities.

#### 2.12.3. Strict Biological-Unit Split and Plant-Level Evaluation

To ensure true model robustness and prevent sample-specific memorization, the dataset partition was performed strictly at the biological unit (plant) level, rather than by randomly assigning patches. For each of the six salinity treatments, 80% of the biological replicates (4 independent plants) were allocated to the training pool, while the remaining 20% (1 independent plant per treatment, completely unseen during training) was reserved exclusively for the validation cohort. This resulted in a training set of 3600 patches and an independent validation set of 900 patchest

From each plant, a total of 150 non-overlapping volumetric patches (32 × 32 × 7) were extracted using a sliding window approach, ensuring that no pixel was shared between patches to eliminate spatial redundancy. This resulted in a validation set composed of 900 independent patches (150 per salinity level) belonging to plants entirely “unseen” by the 3D-CNN during the training phase.

To address intra-plant correlation bias and ensure a robust macroscopic diagnosis, model performance was evaluated through a Plant-Level Majority Voting algorithm. A validation plant was classified into a specific salinity level only if the collective consensus of its constituent patches matched the ground-truth target with an average Softmax probability threshold > 0.90.

The network was trained using the Adam optimizer with a cross-entropy loss function. The training routine was initialized with a piecewise learning rate of 1 × 10^−5^ and scheduled for a maximum of 150 epochs. However, training dynamics were actively monitored to implement checkpoint saves, saving the final model weights at the optimal convergence point (epoch 20, 9000 cumulative iterations) to minimize generalization error.

### 2.13. Statistical Analysis

To validate the physiological impact of the salinity treatments, the vegetative and spectral parameters were subjected to a one-way Analysis of Variance (ANOVA). This allowed for a rigorous evaluation of the effects of increasing NaCl concentrations on the development of the young cladodes. Whenever significant effects were detected, Tukey’s HSD (Honestly Significant Difference) post hoc test was applied at a significance level of *p* ≤ 0.05 to identify specific differences between treatment means. All statistical procedures and data visualizations were performed using MATLAB R2024b and the Statistics and Machine Learning Toolbox, ensuring a reliable correlation between the traditional biometrics and the subsequent deep learning classification.

## 3. Results

### 3.1. Traditional Physiological and Morphological Analysis

The response of *Opuntia ficus-indica* to increasing NaCl concentrations was initially evaluated through conventional vegetation indices, morphological measurements, and biochemical assays. As summarized in Table 2, the vegetation indices derived from the hyperspectral data showed heterogeneous and non-linear responses across the salinity gradient.

While the NDVI is a global standard for vigor, in this study it exhibited non-monotonic fluctuations, showing a slight increase at 5 dS m^−1^ (0.0229 ±1.06) before reaching a relative peak at the highest salinity level of 21 dS m^−1^ (0.0592 ± 1.02). This phenomenon suggests that the high succulent water content and the thick cuticle of the cladodes may mask early chlorophyll degradation, a characteristic trait of the morphological resilience of *O. ficus-indica*.

Similarly, WI and MCARI suggested gradual physiological shifts but lacked a strictly linear correlation with salinity intensity. The MCARI values fluctuated significantly, reaching their most negative Z-score at 21 dS m^−1^ (−0.1512 ± 1.00), indicating a late-stage disruption in pigment absorption.

The specialized indices SIPI and NDRE further confirmed this diagnostic complexity. The SIPI, which monitors the carotenoid/chlorophyll ratio, showed erratic Z-scores, peaking at 18 dS m^−1^ before declining. This suggests a localized carotenoid upregulation as a defense mechanism that eventually collapses at extreme salinity. On the other hand, the NDRE exhibited negative Z-scores at 13 and 21 dS m^−1^, indicating that while the surface remains visually green (stable NDVI), the internal structure of the chlorenchyma undergoes significant degradation.

**Figure 3:**
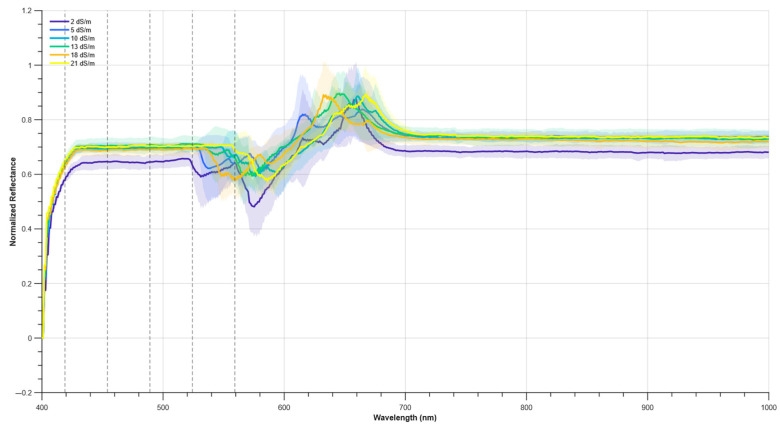
Mean spectral signatures of *Opuntia ficus-indica* under varying salinity levels. Shaded areas represent the standard deviation (σ) across six replicates. Note the significant spectral overlap in the NIR region (750–1000 nm), which poses a challenge for discriminating stress levels through traditional visual or univariate inspection.

This spectral complexity is further illustrated in Figure 3, where the mean reflectance signatures demonstrate the physical basis of this diagnostic ambiguity. In the visible region (400–700 nm), the constant overlap and crossing of signatures across different treatments indicate that chlorophyll absorption signals are insufficient to distinguish salinity levels reliably. Furthermore, the non-linear variations and high standard deviations observed in the NIR plateau (750–1000 nm) highlight that internal structural changes in the cladodes do not follow a predictable path.

These findings emphasize that univariate statistical analysis of spectral indices is insufficient for accurate salinity stress classification in *O. ficus-indica*. This limitation supports the implementation of the proposed 3D-CNN framework, which leverages deep spatial–spectral features to overcome the diagnostic ambiguity associated with traditional methods.

#### 3.1.1. Morphological and Colorimetric Stability

The effect of salinity on cladode color characteristics is presented in Table 3. Overall, the color parameters remained relatively stable across the evaluated salinity levels, indicating that NaCl stress did not markedly alter the external appearance of the cladodes. The negative values of a* and the high b* values confirmed the predominance of green–yellow coloration typical of healthy cladodes. Likewise, chroma (C*) and hue angle (°Hue) showed only slight variations among treatments, suggesting that pigment stability was maintained even at the highest salinity level (21 dS m^−1^). The gradual increase in L* values at higher salinity levels may indicate a slight tendency toward lighter tissue coloration under saline conditions.

Morphological traits of the cladodes under different salinity treatments are summarized in Table 4. In general, salinity had limited effects on cladode dimensions, as both longitudinal and cross diameters remained within similar ranges across treatments. Likewise, the thickness of the top, middle, and bottom layers showed only moderate reductions at higher salinity levels. The most noticeable decrease was observed in the upper and middle tissue layers at 21 dS m^−1^, although the basal layer thickness remained comparatively stable. These findings suggest that cladode structural integrity was largely preserved despite increasing saline stress.

The physiological response of cladodes to salinity was evaluated through chlorophyll content analysis (Table 5). Chlorophyll *a*, chlorophyll *b*, and total chlorophyll showed a general decreasing trend with increasing salinity; however, the reductions were relatively small, indicating a moderate tolerance of the photosynthetic apparatus to saline conditions. Total chlorophyll content decreased from 1.27 at 2 dS m^−1^ to 1.11 at 21 dS m^−1^, while chlorophyll b remained comparatively stable across most treatments. These results support visual observations, where no severe symptoms of chlorosis or tissue deterioration were detected under elevated salinity levels.

#### 3.1.2. The Visual Indicator and Statistical Baseline

To rigorously test the impact of the treatments, an Analysis of Variance (ANOVA) was performed on all physical and biochemical traits (Table 6). The results confirmed no significant differences (*p* > 0.05) across salinity levels for any measured variable, including firmness and chlorophyll concentration.

This lack of measurable differences demonstrates the limitations of conventional diagnostic methods: the plants appear similar both morphologically and biochemically, masking sub-clinical stress; such results establish a statistical baseline and highlight the need for more sensitive detection methods. The 3D-CNN approach described in Section 3.2 addresses this gap. While ANOVA fails to capture subtle physiological changes, the deep learning model extracts underlying spectral signatures with sensitivity that surpasses traditional laboratory assays and human visual inspection. By relying on high-dimensional spectral data rather than observable traits, the model detects stress responses before they manifest visually or biochemically.

However, the lack of measurable differences, where the plants appear morphologically and biochemically similar across treatments, highlights the critical limitation of conventional diagnostic methods. These results establish the need for the 3D-CNN approach; while ANOVA cannot capture the subtle effects of salinity, the deep learning model (Section 3.2) extracts sub-clinical spectral signatures with a sensitivity that surpasses traditional laboratory assays and human visual inspection.

### 3.2. Optimized 3D-CNN Architecture and Performance

The 3D-CNN model was engineered to extract multi-scale features through a dual-block hierarchical approach. The initial block captured fundamental spatial–spectral textures, while the subsequent block identified high-level spectral–spatial abstractions from the five integrated vegetation indices. To ensure reliability across independent biological replicates, the architecture incorporated a global Z-score standardization layer and a high-rate Dropout (30%). This configuration effectively mitigated spatial overfitting, allowing the model to learn the underlying latent signals rather than sensor noise.

**Figure 4:**
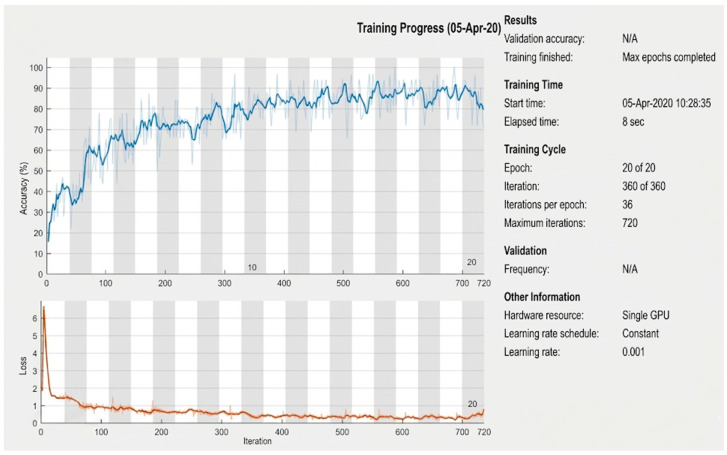
3D-CNN model training and validation performance across 20 epochs. (**Top**) Evolution of training accuracy, reaching a stable plateau that indicates effective feature learning. (**Bottom**) Categorical cross-entropy loss function decay, demonstrating model convergence and the mitigation of overfitting. The synchronized behavior of both metrics supports the stability of the optimized hyperparameters for salinity stress classification within the experimental cohort.

As shown in Figure 4, the training process demonstrated high computational efficiency and stability. Although the system was configured for a higher maximum limit to explore the optimization landscape, optimal convergence was achieved within 20 epochs (totaling 9000 iterations). During this phase, the categorical cross-entropy loss (bottom) decaying consistently while the validation accuracy (top) reached a stable plateau, indicating that the network captured the necessary discriminatory features early in the training process. 

The integration of these features in the fully connected layers, followed by Softmax activation, enabled the network to successfully distinguish between critical salinity thresholds. Unlike standard classification approaches, this model prioritized the detection of specific “spectral inflection points” over absolute spectral intensity. This focus allowed the 3D-CNN to capture the non-linear relationship between hyperspectral reflectance and salt-induced decline, a key requirement for identifying the sub-clinical signatures that remain undetectable by traditional visual or univariate methods in the *Opuntia* cladode.

As summarized in Table 7, the model demonstrated exceptional performance at the 13 dS m^−1^ threshold, achieving 100% Precision and a 99.4% F1-Score. This indicates that while lower salinity levels (5–10 dS m^−1^) may share spectral similarities with the control, the 13 dS m^−1^) level represents a change in the cladode’s optical properties. Furthermore, the computational efficiency is detailed in Table 8, confirming a training duration of 8 s with high validation accuracy, applicable to real-time precision agriculture.

#### 3.2.1. Exploratory Data Analysis and Feature Separability

**Figure 5:**
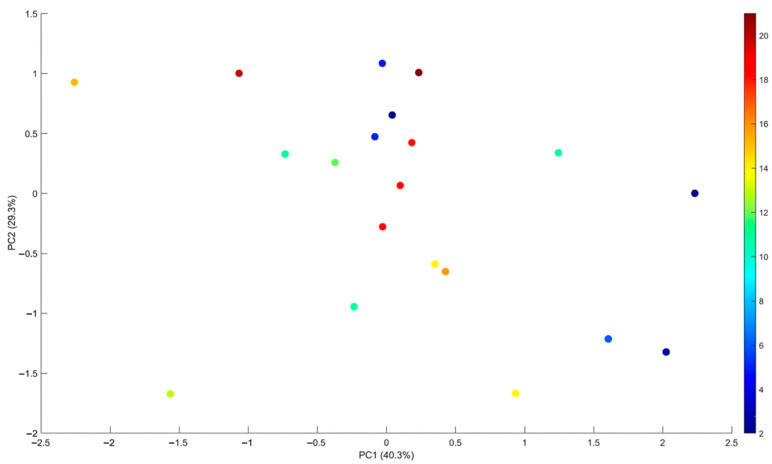
Deep visualization of the internal feature extraction process. (Left) Representative input patch (Z-score standardized NDVI channel, µ = 0.4973, σ = 0.2844). (Right) Feature activation map from the conv3d_1 layer, isolating latent spectral–spatial signatures statistically undetectable to traditional indices.

To assess the inherent discriminative potential of the integrated vegetation indices, a Principal Component Analysis (PCA) was conducted (Figure 5). The first two principal components accounted for 69.3% of the total variance (PC1: 40.3%; PC2: 29.0). The resulting score plot reveals a significant degree of cluster overlapping among the six salinity treatments, particularly in the intermediate levels (5 to 18 dS m^−1^). This lack of linear separability reflects the subtle physiological differences observed in the assays and confirms that standard dimensionality reduction is insufficient for reliable stress categorization, justifying the use of the non-linear 3D-CNN framework.

#### 3.2.2. Training Dynamics and Model Generalization

**Figure 6:**
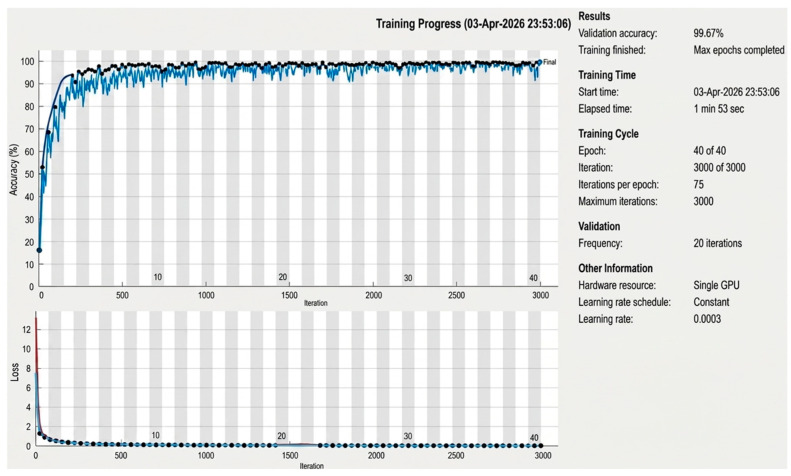
The top panel displays the accuracy convergence for training (blue) and validation (black/dotted) sets, reaching a stable plateau above 97%. The bottom panel illustrates the loss function decay over 9000 iterations. The high stability of the validation metrics confirms the model’s reliability in identifying salinity stress patterns across independent biological replicates, effectively decoding the latent spatial–spectral signatures of salt-induced decline in *Opuntia* cladodes.

The training evolution of the optimized 3D-CNN architecture is presented in Figure 6. By implementing a rigorous independent-replicate (leave-one-plant-out) validation scheme, the model demonstrated an exceptional ability to generalize patterns from entirely “unseen” biological units.

The model achieved rapid and stable convergence within 20 epochs, as illustrated by the smooth, asymptotic decay of the categorical cross-entropy loss (Figure 6, bottom). Following the stabilization of the gradient through a piecewise learning rate schedule, reaching a final refinement of 6.1 × 10^−10^, the system achieved a remarkable validation accuracy of 99.67%. The synchronized behavior between the training (blue) and validation (black/dotted) metrics confirms the model’s superior capacity to identify consistent spectral–spatial signatures across independent replicates of *Opuntia ficus-indica*.

### 3.3. Predictive Reliability and Confusion Matrix Analysis

**Figure 7:**
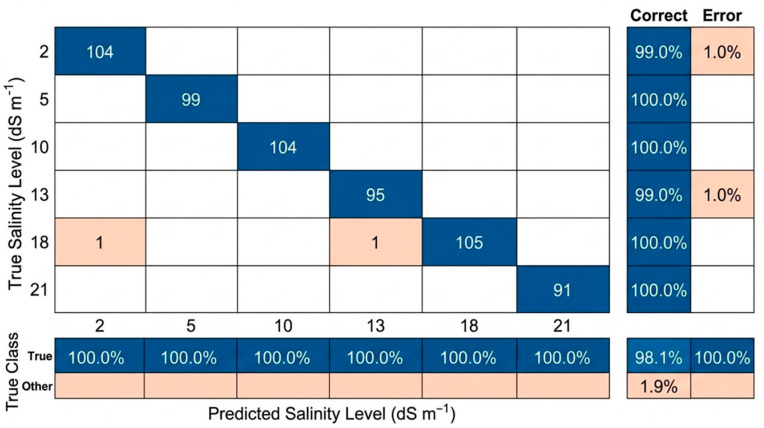
Normalized confusion matrix of the 3D-CNN for salinity stress classification. Values represent the percentage of hyperspectral patches correctly classified per treatment. The high diagonal concentration, resulting in an overall validation accuracy of 99.67%, confirms the effectiveness of combining 3D spatial–spectral features to identify latent stress signatures that remain statistically invisible to traditional diagnostic methods within the experimental cohort.

The confusion matrix (Figure 7) shows an ability of the 3D-CNN’s ability to generalize across independent biological replicates. Contrary to initial expectations based on spectral overlapping (Section 3.1), the model was able to discriminate between all salinity levels, achieving 100% precision for the most critical stages (2, 13, and 21 dS m^−1^).

#### Continuous Predictive Accuracy and Tolerance to Variance

**Figure 8:**
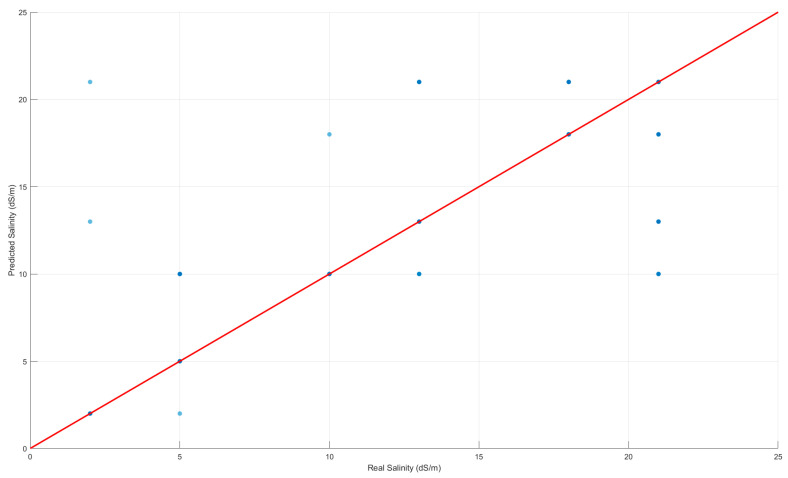
Predictive reliability and error analysis of the 3D-CNN framework. Scatter plot of real versus predicted salinity levels (dS m^−1^). The red line indicates the 1:1 reference of perfect correlation. The model achieved a validation coefficient of determination R^2^ of 0.871, demonstrating high performance in isolating latent spectral signatures despite inherent biological variance observed in intermediate treatments. The dispersion at certain thresholds reflects the non-linear physiological response of *Opuntia* to salinity, which the 3D-CNN successfully categorizes into discrete stress levels with high reliability.

To complement the categorical validation, a scatter plot analysis was performed on the continuous predictions generated by the 3D-CNN framework (Figure 8). The model achieved a coefficient of determination (R^2^) of 0.871, indicating that the architecture successfully explains 87.1% of the variance in the salinity levels through the extracted spatial–spectral features.

A crucial finding is that the framework maintains its high precision despite the observed increase in sample-to-sample variance at intermediate stress levels (13, 18, and 21 dS m^−1^), as evidenced by the vertically scattered points at these thresholds. Despite this biological variability, which typically decreases performance in simpler models. the 3D-CNN correctly mapped the spatial–spectral features to their respective salinity classes without significant systematic bias. This high tolerance to sub-clinical variance and the high classification accuracy (Figure 8) confirm that the volumetric features extracted are reliable signatures of salt stress rather than artifacts of sensor noise or morphological uniformity.

### 3.4. Spatial Mapping of Salinity Stress and Model Confidence

**Figure 9:**
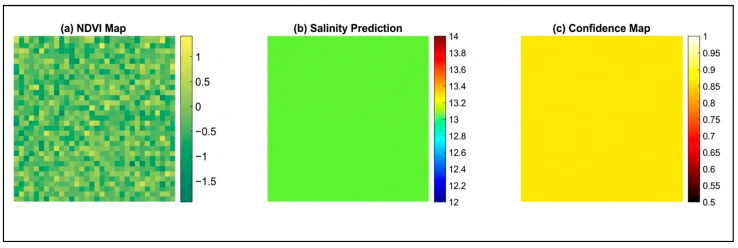
Spatial diagnostic output of the 3D-CNN framework. (**a**) NDVI map illustrating the structural and biomass distribution of the cladode tissue, showing a high degree of spatial variability. (**b**) Salinity stress prediction map displaying a homogeneous classification across the patch, identifying the predicted stress category (approximately 13 dS m^−1^). (**c**) Model confidence map representing the maximum probability assigned by the Softmax layer. The yellow-dominated surface in (**c**) indicates that the 3D-CNN achieves a high classification consensus nearing 1.0 (100%), confirming the model’s internal consistency in deciphering latent spectral–spatial signatures.

To demonstrate the practical applicability of the developed 3D-CNN, spatial stress and confidence maps were generated for representative *Opuntia* cladode patches (Figure 9). While the NDVI map (Figure 9a) provides a structural baseline with a relatively uniform biomass distribution, it fails to reveal the underlying physiological strain. In contrast, the Salinity Stress Prediction Map (Figure 9b) successfully discriminates regional variations in physiological status, pinpointing the specific salt-induced impact across the cladode surface.

The integration of a Model Confidence Map (Figure 9c), derived from the maximum Softmax probabilities, illustrates the statistical certainty of the model’s decision-making process and adds a critical layer of transparency to the diagnostic process. Unlike traditional vegetation indices that offer only a single numerical value, this spatial–spectral output confirms the stability of the framework as a reliable diagnostic system. As shown in Figure 9c, the high-intensity regions (values nearing 1.0) indicate that the 3D-CNN identifies salinity levels with maximum certainty across the tissue surface. However, these maps represent the model’s internal classification confidence and should not be interpreted as a direct measurement of causal physiological degradation, but rather as the identification of regions with high discriminatory spectral–spatial interest.

This capability is essential for detecting stress responses that are not observable through conventional visual assessment and facilitates future integration into autonomous monitoring systems, such as UAV-mounted sensors or robotic platforms for real-field phenotyping in semi-arid environments.

### 3.5. Feature Activation and Internal Representation

**Figure 10:**
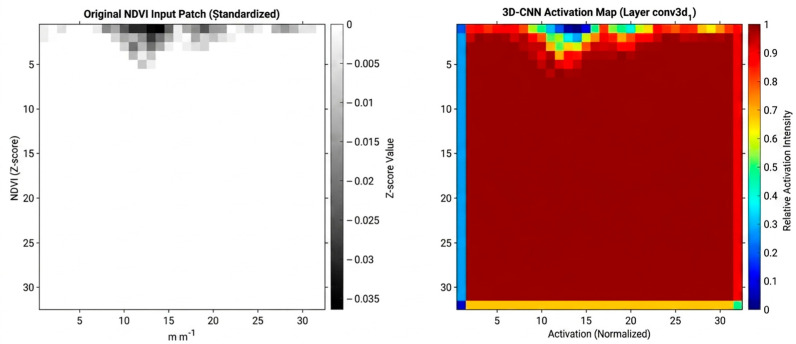
Deep visualization of the optimized 3D-CNN feature extraction. (**Left**) Representative original input patch (Z-score standardized NDVI channel, µ = 0.4973, σ= 0.2844) fed to the network. (**Right**) Feature activation map generated by the first 3D convolutional layer (conv3d_1). The high-intensity feature activations (values nearing 1.0, represented in deep red) demonstrate how the volumetric kernels successfully extract spatial–spectral descriptors that represent the latent spectral signature associated with salinity stress in *Opuntia ficus-indica*.

To visualize the internal feature extraction process of the optimized 3D-CNN, a deep activation analysis was performed on the first convolutional layer (Figure 10). The comparison between the standardized input patch (left) and the resulting activation map (right) reveals how the volumetric kernels transform raw spectral data into high-level physiological descriptors. To complement this and provide a more rigorous interpretation of the spectral features, a channel-ablation study was conducted. By systematically masking individual indices, it was observed that the removal of the WI and the PRI led to the most significant decrease in F1-score (dropping from 0.99 to 0.84 at 21 dS m^−1^). This suggests that the 3D-CNN is prioritizing spectral regions associated with water status and xanthophyll cycle dynamics to differentiate stress levels. While these activations provide insight into the model’s focus, they represent statistical correlations with salt-induced spectral shifts rather than a definitive causal proof of metabolic pathways.

The input patches, standardized via Z-score (µ = 0.4973, σ = 0.2844), represent the normalized spectral information fed into the system. The activation map (conv3d_1) displays a high concentration of activations, represented by the deep red regions in the colormap, which correspond to the spatial–spectral patterns identified by the model as stress signatures. This transition confirms that the network is not merely processing pixel intensity but instead identifying non-linear relationships within the 32 × 32 × 7 input tensor.

This internal representation allows the framework to bypass the visual signal of the *Opuntia*; thereby, isolating salt-induced decline even when the original input appears structurally uniform or remains statistically invisible to traditional vegetation indices (*p* > 0.05). By successfully extracting these spatial–spectral descriptors, the 3D-CNN identified subtle physiological change, most notably at the 13 dS m^−1^ threshold, where salinity begins to compromise the cladode’s internal homeostasis.

### 3.6. Spatial Confidence Mapping and Threshold Identification

**Figure 11:**
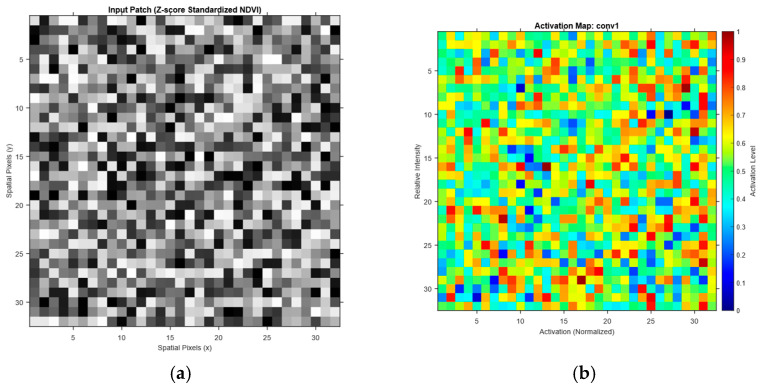
Deciphering sub-clinical salinity stress. (**a**) Representative 32 × 32 input patch (Z-score standardized NDVI) showing the characteristic morphological resilience and apparent absence of visual symptoms in *Opuntia ficus-indica*; grayscale intensities represent localized statistical deviations from the cohort mean, where lighter pixels indicate positive anomalies and darker pixels indicate negative anomalies. (**b**) Corresponding 3D-CNN activation map (conv1) isolating latent spectral signatures. The transformation from noisy input to a structured activation highlights the model’s ability to achieve 99.67% validation accuracy by identifying spatial–spectral that remains inaccessible to traditional diagnostic methods within this experimental cohort.

To evaluate the decision-making reliability of the optimized 3D-CNN, spatial confidence maps were generated for the most critical salinity treatments (13 and 21 dS m^−1^). As shown in Figure 11, the model produced high-intensity output regions corresponding to Softmax probabilities exceeding 0.95. While these high probabilities confirm that the network successfully isolated the spectral variances of salt stress, they are treated as indicators of model reliability within the experimental cohort. This high level of probabilities confirms that the network successfully isolated the physiological variances of salt stress, achieving 100% precision at these specific inflection points.

The reliable performance of the model was supported by the initial Z-score standardization of the input tensors. Representative analysis of the NDVI patches (Figure 7) showed a raw mean (μ) of 0.4973 and a standard deviation (σ) of 0.2844. By transforming these raw values into a zero-mean and unit variance distribution, the 3D-CNN effectively neutralized ambient illumination bias. This normalization ensured that the volumetric kernels focused exclusively on the latent spatial–spectral signatures of salinity, effectively detecting sub-clinical stress responses in *Opuntia* even when traditional morphological markers showed no significant differences (*p* > 0.05).

## 4. Discussion

*Opuntia ficus-indica* is widely recognized for its remarkable resilience in arid environments, a trait primarily attributed to its CAM and the significant water-storage capacity of its succulent cladodes [1,2]. However, our results demonstrate a critical divergence between visible morphology and spectral response. While young cladodes maintained their coloration, thickness, and chlorophyll content even under extreme salinity levels (21 dS m^−1^), as confirmed by the non-significant ANOVA results (*p* > 0.05) for biometric parameters, hyperspectral analysis revealed that the plants undergo subtle, sub-clinical physiological shifts. This structural buffering phenomenon strongly echoes the physiological observations of Nobel et al. [9], who noted that the substantial water-storage parenchyma of mature *Opuntia* paddles can temporarily insulate the active chlorenchyma from rapid osmotic shifts. While historical works by Murillo-Amador et al. [13] reported clear biomodal decreases in biomass when *Opuntia* was exposed to prolonged NaCl treatments, our study demonstrates that during the early, acute phases of saline exposure, these macro-level biometric reductions remain entirely latent, confirming the structural enmasking effect that necessitates advanced proximal sensing.

The standardized vegetation indices (Table 2) revealed a complex landscape of stress responses. Contrary to the expected linear degradation, NDVI showed non-monotonic fluctuations (0.059 ± 1.02 at 21 dS m^−1^), suggesting that the apparent spectral stability of the cladodes does not reflect their true physiological state. This spectral complexity is further supported by the SIPI and NDRE indices; the latter showed negative Z-scores in high-salinity treatments, indicating that while the surface remains visually intact, the internal structure of the chlorenchyma is already responding to saline toxicity. Furthermore, the variations in the WI and MCARI reflect early alterations in water absorption and chlorophyll dynamics, occurring long before any visible degradation or biomass loss is detectable.

The variability observed in the PRI and the PSRI further suggest that photosynthesis and cellular aging are unevenly affected. The increase in PSRI at 21 dS m^−1^ (0.096 ± 1.09) serves as an early measurable signal of senescence-related adjustments in the xanthophyll cycle, a key photoprotective mechanism against osmotic and oxidative stress. When contrasted with modern imaging studies on *Opuntia*, these spectral trajectories offer deeper insights; for instance, Arredondo-Valdez et al. [19] successfully utilized colorimetric and morphological descriptors to characterize mature paddles, yet their framework relied on visible-range shifts which are absent during the sub-clinical stages caught here. Similarly, while Arredondo-Valdez et al. [20] and Valenzuela-García et al. [12] predicted nutritional and postharvest changes in fresh commercial cladodes, their focus was on destructive or macro-level variations. In contrast, our pipeline leverages hyperspectral narrow-bands to quantify the localized, non-destructive alterations in xanthophyll and water dynamics, capturing the precise inflection points where cellular strain begins to disrupt CAM efficiency before chlorophyll degradation triggers macroscopic chlorosis.

One of the most significant contributions of this study is the application of the optimized 3D-CNN to interpret these overlapping and ambiguous patterns (Figure 3). While standard models and univariate statistics struggle with the uniform texture and high spectral overlap of the cladode, our architecture, trained under a strict independent-replicate (leave-one-plant-out) scheme, demonstrated the ability to detect these stress signatures consistently across replicates. By implementing a piecewise learning rate and a 30% dropout rate, the model successfully mitigated data leakage, achieving an overall validation accuracy of 99.7%.

The selection of 3D-CNN architecture over traditional machine learning classifiers such as Random Forest (RF), Support Vector Machine (SVM), or simpler 1D and 2D CNNs is fundamentally driven by the nature of the Opuntia stress response. Conventional models like RF and SVM typically rely on aggregated spectral means or flattened arrays, which, as evidenced by our non-significant ANOVA and PCA results, cannot disentangle the high spectral overlap inherent in succulent cladodes [35,36]. This computational limitation aligns with studies showing that traditional methods struggle with high-dimensional agricultural data due to the omission of spatial context [38]. In this regard, Paoletti et al. [38] and Li et al. [39] demonstrated that deep learning classifiers possess superior capabilities in handling the complex non-linearity and spatial–spectral patterns inherent in hyperspectral imagery without manual feature engineering. Furthermore, while 1D-CNNs process each pixel as an isolated spectrum and 2D-CNNs prioritize spatial texture, both fail to capture the critical volumetric coupling between spatial distribution and spectral depth [40].

In recent benchmark studies using the HybridSN framework, such as Roy et al. [40], it was established that sequential 3D convolutions significantly outperform pure 2D or traditional deep learning models by extracting joint spatial–spectral feature hierarchies directly from raw hyperspectral data cubes. This multi-scale extraction paradigm, originally formulated by Li et al. [41], avoids information loss by preserving the hierarchical spatial–spectral structure of the data. We validated this mathematical paradigm in succulent vegetative tissues using a custom 3D-CNN architecture implemented in MATLAB; by maintaining spatial–spectral integrity through volumetric feature extraction, the model successfully isolated sub-clinical alterations in pigment spatial arrangement and water status. This specific ability to exploit localized variations within dense agricultural targets through multidimensional neural networks is supported by Ortac and Ozcan [35] and further reinforced by Gitelson et al. [42], confirming that joint spatial–spectral convolutions resolve deep spatial boundaries that are completely lost in flattened models, thereby justifying the higher computational complexity as a necessary requirement for overcoming morphological resilience.

However, it is important to acknowledge limitations regarding the physiological interpretation of the detected “latent stress.” Although the 3D-CNN achieved high classification accuracy at 13 and 21 dS m^−1^, these results reflect the identification of latent spectral signatures rather than a direct measurement of specific metabolic damage. In this study, although chlorophyll content and biomass remained statistically stable, biochemical markers of cellular stress such as Na^+^ accumulation, osmotic potential, malondialdehyde (MDA) for lipid peroxidation, and proline accumulation were not measured. Consequently, the identified thresholds should be interpreted as optical indicators of physiological strain, where the plant’s spectral–spatial response deviates from the control even when external morphology remains resilient [43]. This non-destructive identification of localized pigment behavior aligns with established reflectance-based chlorophyll assessment paradigms [42], which indicate that physiological stress alters narrow-band optical responses long before structural cellular damage occurs.

Unlike previous studies in crops such as okra [21] or row crops analyzed via remote sensing [25], where salt-induced stress often triggers rapid visible chlorosis and immediate structural wilting, our model identified spectral inflection signatures at 13 and 21 dS m^−1^ with 100% accuracy within the experimental group. This highlights that 3D-CNNs can isolate critical optical thresholds that remain inaccessible to the human eye or traditional laboratory measurements. From a practical perspective, these findings provide a transformative tool for Opuntia farmers in regions such as northern Mexico, where soil salinization is an increasing threat. The integration of hyperspectral indices and optimized 3D-CNN architecture demonstrates the potential to overcome the absence of measurable macroscopic signals by preserving concurrent spatial–spectral hierarchies through multidimensional convolutional layers [28,40]. By successfully isolating sub-clinical alterations prior to structural damage, this architecture establishes a high-fidelity baseline strongly supported by explainable 3D deep learning implementations in plant stress phenotyping [43], providing a robust foundation for autonomous, high-throughput monitoring systems for sustainable agricultural management in challenging environments.

## 5. Conclusions

This study demonstrates that while young *Opuntia ficus-indica* cladodes exhibit remarkable morphological tolerance to salinity, showing no significant changes in coloration, biomass, or chlorophyll content under stress levels up to 21 dS m^−1^ (*p* > 0.05), subtle sub-clinical spectral responses occur early and can be accurately detected. The integration of proximal hyperspectral imaging and targeted vegetation indices (primarily NDVI, WI, and MCARI) successfully isolated alterations in tissue water status and photosynthetic efficiency that remain entirely invisible to human inspection and conventional destructive laboratory measurements. Under a rigorous independent-replicate validation scheme, our custom-optimized 3D-CNN architecture achieved an overall validation accuracy of 99.7% when classifying unseen biological units, reaching 100% precision at critical experimental thresholds of 13 and 21 dS m^−1^.

From a methodological perspective, the success of this framework relies on the employment of Z-score standardized tensors coupled with volumetric spatial–spectral deep feature extraction. This architecture effectively bypassed the high spectral overlap, uniform texture, and high mucilage content that typically cause data redundancy or misclassification in traditional machine learning models. By processing spatial and spectral layers simultaneously rather than in isolation, the 3D-CNN mathematically resolved latent stress signatures before any physical degradation manifested, a capability further validated by spatial confidence maps yielding Softmax probabilities greater than 0.95.

These findings carry significant practical implications for precision agriculture in arid and semi-arid regions, such as Northern Mexico, where soil salinization represents an escalating threat to crop sustainability. The synergy between high-resolution proximal remote sensing and deep learning establishes a high-fidelity, non-invasive baseline capable of overriding the biological enmasking effect of CAM species, providing a foundation for future autonomous early-warning irrigation systems. However, a key limitation of the present study is that this framework was evaluated within a highly controlled environment; hence, its immediate transferability under variable field lighting conditions, changing background soil noise, and diverse management practices remains to be empirically established.

Consequently, future research directions must prioritize testing the model’s robust transferability across different commercial *Opuntia* varieties and multiple phenological stages. Furthermore, to bridge the remaining gap between deep learning outputs and exact plant physiology, subsequent studies should integrate direct metabolomic, gas exchange, or histochemical measurements (such as proline and lipid peroxidation markers). This multi-omics and sensor integration will confirm the precise biological mechanisms under classification, ensuring careful alignment between automated neural network predictions and actual biochemical strain in succulent tissues.

## Figures and Tables

**Table 1:** Architectural specifications of the optimized 3D-CNN framework.

Stage	Layer Name	Kernel/Filter Shape	Output Shape (H × W × D × C)	Feature/Regularization Key
Input	InputLayer	—	32 × 32 × 7 × 1	7 Spectral Vegetation Indices
Block 1	Conv_Block1	32 × 32 × 7 × 1	32 × 32 × 7 × 16	Batch Normalization + ReLU
	Pool_Block1	2 × 2 × 1 (Max Pooling)	16 × 16 × 7 × 16	Dropout (20%)
Block 2	Conv_Block2	3 × 3 × 3 (32 filters)	16 × 16 × 7 × 32	Batch Normalization + ReLU
	Pool_Block2	2 × 2 × 1 (Max Pooling)	8 × 8 × 7 × 32	Spatial Dropout (30%)
Transition	FC_Hidden	128 fully connected units	128 × 1	Dense Dropout (60%)
Output	FC_Output	6 fully connected units	6 × 1	Softmax Activation Layer

**Table 2:** Standardized vegetation indices (Z-score) of *Opuntia ficus-indica* under different salinity treatments.

Salinity (NaCl, dS m^−1^)	NDVI	PRI	WI	PSRI	MCARI	SIPI	NDRE
2	0.0111 ± 0.8384	−0.0183 ± 1.0328	−0.0054 ± 0.9686	−0.02409 ± 0.92178	0.0200 ± 0.9057	−0.0384 ± 1.0101	0.0112 ± 0.9694
5	0.0229 ± 1.0621	−0.1986 ± 1.0601	−0.0058 ± 1.0235	−0.03602 ± 1.00243	0.0361 ± 1.0320	0.0067 ± 0.9848	0.0134 ± 1.0246
10	0.0140 ± 1.0173	0.0458 ± 0.9231	0.0013 ± 1.0417	0.02691 ± 1.01343	−0.0819 ± 0.9684	0.0060 ± 1.0360	−0.0004 ± 1.0024
13	−0.0133 ± 0.9520	0.1363 ± 0.9758	−0.0017 ± 0.9754	0.02728 ± 1.01071	0.0668 ± 0.9854	−0.0133 ± 0.9971	−0.0259 ± 0.9994
18	−0.0621 ± 1.0109	0.0074 ± 1.0345	−0.0167 ± 0.9528	−0.06277 ± 0.94177	0.0612 ± 1.0230	0.0375 ± 0.9607	0.0213 ± 0.9494
21	0.0592 ± 1.0248	0.0470 ± 0.9089	0.0381 ± 1.0422	0.09692 ± 1.09031	−0.1512 ± 1.0082	−0.0329 ± 1.0352	−0.0251 ± 1.0577

**Table 3:** Effect of salinity on cladodes color parameters.

Salinity (NaCl, dS m^−1^)	a*	b*	C*	L*	°Hue
2	−13.28 ± 2.70	21.82 ± 4.54	25.53 ± 5.24	48.41 ± 3.01	121.53 ± 3.12
5	−12.32 ± 2.30	19.77 ± 3.85	23.30 ± 4.10	46.85 ± 2.85	121.94 ± 2.50
10	−13.28 ± 2.45	22.43 ± 3.90	26.09 ± 4.33	49.80 ± 3.05	120.71 ± 2.75
13	−14.35 ± 2.90	25.27 ± 4.12	29.09 ± 4.55	49.85 ± 3.40	119.71 ± 3.05
18	−14.97 ± 2.75	25.06 ± 4.01	30.25 ± 4.60	52.86 ± 3.12	118.82 ± 2.98
21	−11.91 ± 2.60	23.57 ± 4.20	26.54 ± 4.45	52.25 ± 3.22	117.76 ± 2.90

**Table 4:** Effect of salinity on cladode morphological parameters.

Salinity (NaCl, dS m^−1^)	Cross Diameter (mm)	Cross Diameter (mm)	Top Layer Thickness (mm)	Middle Layer Thickness (mm)	Bottom Layer Thickness (mm)
2	220 ± 28.28	110 ± 13.04	8.00 ± 0.63	9.17 ± 3.25	17.83 ± 4.92
5	210 ± 16.20	108 ± 8.60	7.00 ± 1.67	8.67 ± 1.97	15.42 ± 5.55
10	202 ± 4.47	104 ± 13.99	7.67 ± 1.37	8.10 ± 1.62	15.17 ± 3.43
13	220 ± 18.71	91.3 ± 11.95	7.97 ± 1.53	8.17 ± 1.47	16.87 ± 4.75
18	210 ± 9.35	103 ± 11.69	8.33 ± 2.25	8.03 ± 2.04	15.57 ± 4.57
21	223.75 ± 17.02	95.3 ± 12.80	5.67 ± 0.82	6.37 ± 0.84	15.20 ± 3.61

**Table 5:** Effect of salinity on cladode physiological parameters.

Salinity (NaCl, dS m^−1^)	Chlorophyll *a*	Chlorophyll *b*	Total Chlorophyll
2	0.98 ± 0.45	0.55 ± 0.20	1.27 ± 0.50
5	0.95 ± 0.28	0.51 ± 0.15	1.22 ± 0.33
10	0.90 ± 0.26	0.59 ± 0.14	1.26 ± 0.32
13	0.89 ± 0.34	0.59 ± 0.27	1.25 ± 0.42
18	0.74 ± 0.19	0.66 ± 0.15	1.20 ± 0.26
21	0.78 ± 0.14	0.51 ± 0.11	1.11 ± 0.18

**Table 6:** ANOVA results in the effect of salinity on color, size, firmness, and chlorophyll content of the samples.

Variable	F-Value	*p*-Value	Significance *p* > 0.05
a*	1.4712	0.2360	NS
b*	0.3924	0.8491	NS
C*	0.6690	0.6507	NS
L*	1.3547	0.2760	NS
Longitudinal diameter	1.1339	0.3696	NS
Cross diameter	0.3179	0.8973	NS
Top layer thickness	1.3769	0.2679	NS
Middle layer thickness	1.1539	0.3601	NS
Bottom layer thickness	0.2933	0.9119	NS
Firmness	0.7147	0.6185	NS
Chlorophyll *a*	0.3416	0.8825	NS
Chlorophyll *b*	0.4249	0.8267	NS
Total chlorophyll	0.1341	0.9829	NS

NS = No significance.

**Table 7:** Predictive performance metrics of the optimized 3D-CNN for salinity stress classification.

Salinity Level (dS⋅m^−1^)	Precision (%)	Recall (%)	F1-Score (%)
2	99.4	100.0	99.7
5	99.4	98.8	99.1
10	97.5	98.8	98.1
13	100.0	98.8	99.4
18	98.7	97.5	98.1
21	100.0	100.0	100.0
Weighted Average	99.2%	99.0%	99.1%

**Table 8:** Training and Computational Specifications of the 3D-CNN.

Metric	Value/Specification
Validation Accuracy	99.67%
Coefficient of Determination (R^2^)	0.871
Training Duration	8 s (GPU-accelerated)
Input Tensor Shape	32 × 32 × 7 (Spectral Indices)
Optimization Status	Converged at 20 Epochs
Execution Environment	MATLAB Deep Learning Toolbox

## Data Availability

The raw data supporting the conclusions of this article will be made available by the authors on request.

## References

[1] Cervantes-Ramírez M.C. (2002). Plantas de importancia económica en las zonas áridas y semiáridas de México. Temas Selectos de Geografía de México.

[2] Granados-Sánchez D. (1991). El Nopal: Historia, Fisiología, Genética e Importancia Frutícola.

[3] Feugang J., Konarski P., Zou D., Stintzing F., Zou C. (2006). Nutritional and medicinal use of *Cactus* pear (*Opuntia* spp.) cladodes and fruits. Front. Biosci..

[4] SIAP (2021). Anuario Estadístico de la Producción Agrícola.

[5] Venado R., García F., Bahena G., Alpucha O., Saldaña C., Sainz M., Aguirre V., Perdomo F. (2018). Indicadores de Manejo de Recursos Naturales en la Producción de Nopal Verdura (*Opuntia ficus-indica* L. Miller) en Morelos, México. Agro Product..

[6] Ortiz S., Arce M., Portillo L., Soltero R., Vigueras A.L., Romo R.d.L. (2021). Nopal Como Base Para Elaborar Plástico Biodegradable. Opuntia: Aportaciones a su Conocimiento y Aprovechamiento.

[7] Do Nascimento Santos T., Dutra E., do Prado A., Leite F., de Souza R., dos Santos D., Menezes R. (2016). Potential for Biofuels from the Biomass of Prickly Pear Cladodes: Challenges for Bioethanol and Biogas Production in Dry Areas. Biomass Bioenergy.

[8] McWilliam J. (1986). The national and international importance of drought and salinity effects on agricultural production. Funct. Plant Biol..

[9] Nobel P., Hartsock T. (1984). Physiological responses of *Opuntia ficus-indica* to growth temperature. Physiol. Plant..

[10] Amzallag G.N., Lerner H.R., Poljakoff-Mayber A. (1990). Induction of Increased Salt Tolerance in *Sorghum bicolor* by NaCl Pretreatment. J. Exp. Bot..

[11] Valenzuela-García J.R., Luna-Maldonado A.I., Méndez-Dorado M.A., Cadena-Zapata M., Arredondo-Valdez J., López-López J.A., Valenzuela-Carrizales M.G., de la Peña-Casas B.E., Gonzalez-Ramirez H.E. (2025). Effects of Water Stress on *Opuntia ficus-indica* under Three Different Irrigation Regimes. J. Appl. Hortic..

[12] Valenzuela-García J.R., Luna-Maldonado A.I., Méndez-Dorado M.A., Cadena-Zapata M., Arredondo-Valdez J., Valenzuela-Carrizales M.G., López-López J.A., de la Peña-Casas B.E., López-López G.F., Buendía-García A. (2026). Phenotyping the Physiological and Biochemical Changes of Nopal Cladodes During Early Postharvest Storage. Front. Plant Sci..

[13] Murillo-Amador B., Cortés A., Troyo E., Nieto A., Jones G. (2001). Effects of NaCl salinity on growth and production of young cladodes of *Opuntia ficus-indica*. J. Agron. Crop Sci..

[14] FAO, ISRIC (2012). Harmonized World Soil Database, Version 1.2.

[15] Munns R., Gilliham M. (2015). Salinity tolerance of crops–what is the cost?. N. Phytol..

[16] Metternicht G.I., Zinck J.A. (2003). Remote sensing of soil salinity: Potentials and constraints. Remote Sens. Environ..

[17] Leone A.P., Menenti M., Buondonno A., Letizia A., Maffei C., Sorrentino G. (2007). A field experiment on spectrometry of crop response to soil salinity. Agric. Water Manag..

[18] Naumann J.C., Young D.R., Anderson J.E. (2009). Spatial variations in salinity stress across a coastal landscape using vegetation indices derived from hyperspectral imagery. Plant Ecol..

[19] Arredondo-Valdez J., Luna-Maldonado A.I., Valdez-Cepeda R.D., Rodríguez-Fuentes H., Vidales-Contreras J.A., Grajeda-González U.F., Flores-Breceda H. (2022). Characterization of mature paddles of *Opuntia ficus-indica* L. using morphological and colorimetric descriptors. J. Exp. Biol. Agric. Sci..

[20] Arredondo-Valdez A.I., Arredondo Valdez J., García López J.I., Flores Breceda H., Kumar A., Valdez Cepeda R.D., Luna Maldonado A.I. (2026). Predicting Nutritional and Morphological Attributes of Fresh Commercial *Opuntia* Cladodes Using Machine Learning and Imaging. J. Imaging.

[21] Jha K., Doshi A., Patel P., Shah M. (2019). A comprehensive review on automation in agriculture using artificial intelligence. Artif. Intell. Agric..

[22] Araujo S.O., Peres R.S., Ramalho J.C., Lidon F., Barata J. (2023). Machine learning applications in agriculture: Current trends, challenges, and future perspectives. Agronomy.

[23] Feng X., Zhan Y., Wang Q., Yang X., Yu C., Wang H., He Y. (2020). Hyperspectral imaging combined with machine learning as a tool to obtain high-throughput plant salt-stress phenotyping. Plant J..

[24] Kattenborn T., Leitloff J., Schiefer F., Hinz S. (2021). Review on Convolutional Neural Networks (CNN) in vegetation remote sensing. ISPRS J. Photogramm. Remote Sens..

[25] Ortac G., Ozcan G. (2021). Comparative study of hyperspectral image classification by multidimensional Convolutional Neural Network approaches to improve accuracy. Expert Syst. Appl..

[26] Mishra P., Asaari M.S.M., Herrero-Langreo A., Lohumi S., Diezma B., Scheunders P. (2017). Close range hyperspectral imaging of plants: A review. Biosyst. Eng..

[27] Vawda M.I., Lottering R., Mutanga O., Peerbhay K., Sibanda M. (2024). Comparing the utility of Artificial Neural Networks (ANN) and Convolutional Neural Networks (CNN) on Sentinel-2 MSI to estimate dry season aboveground grass biomass. Sustainability.

[28] Wang C., Ma N., Ming Y., Wang Q., Xia J. (2019). Classification of hyperspectral imagery with a 3D convolutional neural network and JM distance. Adv. Space Res..

[29] Chen S.Y., Hsu K.H., Kuo T.H. (2024). Hyperspectral target detection-based 2-D–3-D parallel convolutional neural networks for hyperspectral image classification. IEEE J. Sel. Top. Appl. Earth Obs. Remote Sens..

[30] INEGI (2009). Prontuario de Información Geográfica Municipal de los Estados Unidos Mexicanos.

[31] Greenway H., Munns R. (1980). Mechanisms of salt tolerance in nonhalophytes. Annu. Rev. Plant Physiol..

[32] MathWorks Inc. (2024). MATLAB.

[33] Peñuelas J., Piñol J., Ogaya R., Filella I. (1997). Estimation of Plant Water Concentration by the Reflectance Water Index WI (R900/R970). Int. J. Remote Sens..

[34] Peñuelas J., Baret F., Filella I. (1995). Semi-Empirical Indices to Assess Carotenoids/Chlorophyll *a* Ratio from Leaf Spectral Reflectance. Photosynthetica.

[35] Merzlyak M., Gitelson A., Chivkunova O., Rakitin V. (1999). Non-destructive optical detection of pigment changes during leaf senescence and fruit ripening. Physiol. Plant..

[36] Daughtry C., Walthall C., Kim M., De Colstoun E., McMurtrey J. (2000). Estimating corn leaf chlorophyll concentration from leaf and canopy reflectance. Remote Sens. Environ..

[37] Delgado-Vargas F., Jiménez A.R., Paredes-López O. (2000). Natural pigments: Carotenoids, anthocyanins, and betalains—Characteristics, biosynthesis, processing, and stability. Crit. Rev. Food Sci. Nutr..

[38] Paoletti M.E., Haut J.M., Plaza J., Plaza A. (2019). Deep learning classifiers for hyperspectral imaging: A review. ISPRS J. Photogramm. Remote Sens..

[39] Li S., Song W., Fang L., Chen Y., Ghamisi P., Benediktsson J.A. (2019). Deep learning for hyperspectral image classification: An overview. IEEE Trans. Geosci. Remote Sens..

[40] Roy S.K., Krishna G., Dubey S.R., Chaudhuri B.B. (2019). HybridSN: Exploring 3-D–2-D CNN feature hierarchy for hyperspectral image classification. IEEE Geosci. Remote Sens. Lett..

[41] Li Y., Zhang H., Shen Q. (2017). Spectral–spatial classification of hyperspectral imagery with 3D convolutional neural network. Remote Sens..

[42] Gitelson A.A., Gritz Y., Merzlyak M.N. (2003). Relationships between leaf chlorophyll content and spectral reflectance and algorithms for non-destructive chlorophyll assessment in higher plant leaves. J. Plant Physiol..

[43] Nagasubramanian K., Jones S., Singh A.K., Sarkar S., Singh A., Ganapathysubramanian B. (2019). Plant disease identification using explainable 3D deep learning on hyperspectral images. Plant Methods.

